# A Machine Learning Approach to Analyze Home Advantage during COVID-19 Pandemic Period with Regards to Margin of Victory and to Different Tournaments in Professional Rugby Union Competitions

**DOI:** 10.3390/ijerph182312711

**Published:** 2021-12-02

**Authors:** Alexandru Nicolae Ungureanu, Corrado Lupo, Paolo Riccardo Brustio

**Affiliations:** 1 Department of Medical Science, University of Turin, 10143 Turin, Italy; alexandru.ungureanu@unito.it; 2 NeuroMuscularFunction|Research Group, School of Exercise & Sport Sciences, University of Turin, 10143 Turin, Italy; paoloriccardo.brustio@univr.it; 3 Department of Neuroscience, Biomedicine and Movement, University of Verona, 37131 Verona, Italy

**Keywords:** COVID-19, home advantage, rugby union, margin of victory, decision trees

## Abstract

Home advantage (HA) is the tendency for sporting teams to perform better at their home ground than away from home, it is also influenced by the crowd support, and its existence has been well established in a wide range of team sports including rugby union. Among all the HA determinants, the positive contribute of the crowd support on the game outcome can be analyzed in the unique pandemic situation of COVID-19. Therefore, the aim of the present study was to analyze the HA of professional high-level rugby club competition from a complex dynamical system perspective before and during the COVID-19 pandemic. HA was analyzed in northern and southern hemisphere rugby tournaments with (2013–2019) and without (2020/21) crowd support by the means of the exhaustive chi-square automatic interaction detection (CHAID) decision trees (DT). HA was mitigated by the crowd absence especially in closed games, although differences between tournaments emerged. Both for northern and southern hemisphere, the effect of playing without the crowd support had a negative impact on the home team advantage. These findings evidenced that in ghost games, where differences in the final score were less than a converted try (7 points), HA has disappeared.

## 1. Introduction

Home advantage (HA) in sport depends on several factors and it should be analyzed from a complex dynamical system perspective. Although HA has been well-documented in several competitive sports (baseball, basketball, handball, indoor soccer, roller hockey, rugby, soccer, volleyball, and water polo) [1], the causes are less well understood [2]. According to Nevill and Holder [3], the factors associated with HA for all sports include crowd support, travel fatigue, familiarity with local conditions, territoriality, referee bias, special tactics, and psychological factors, even if territoriality, referee bias, and other psychological factors are all thought to be influenced by the crowd support [2]. In addition, rugby union provides an important context to explore this phenomenon because of the high level of home advantage [1] and the use of a television match official to help to provide a less biased decision by the referees. HA in rugby union was investigated both in northern [1,4,5,6] and southern hemisphere [7,8]. In particular, HA was reported to be oscillating around 60% in northern hemisphere international competitions between 1883 and 2011 [5] and to have a mean of 7 points in national and international southern hemisphere competition [7,8]. Nevertheless, it varied between teams from season to season [7] and during non-professional era (i.e., before 1995) [5].

Among all the determinants of the HA, the positive contribute of the crowd support on the game outcome can be analyzed in the unique pandemic situation of COVID-19. During the first phase of the COVID-19 pandemic, (i.e., March 2020) several sport competitions started to be suspended and subsequently some of them were resumed, while others were cancelled for the 2019–2020 season [9]. In Europe, the Six Nations Tournament and the main club competitions (i.e., English Premiership, Pro14) were suspended in March 2020 and rescheduled during the following summer, while the club competition in France (i.e., Top 14 2019–2020) was cancelled after the 17th matchday. In the southern hemisphere, the 2020 Super Rugby competition involving teams from Argentina, Australia, Japan, New Zealand and South Africa was cancelled after 46 games, whereas regional tournaments replaced it in New Zealand, Australia, South Africa, and crowds were regularly allowed to attend the tournaments. At a national level, the 2020 National Provincial Championship in New Zealand and the 2020 Currie Cup in South Africa were played but no crowds or limited crowds were allowed, while the National Rugby Championship in Australia was cancelled.

Although the pandemic restrictions was reported to impact HA in rugby [10], the application of a non-linear data mining techniques considering contingencies (e.g., the tournament, the margin of victory, the scoring first) may explore potentially useful information in a large dataset, and produce a simple and understandable message to the stakeholders [11]. In fact, since the game outcome is affected by the location (i.e., home or away), the margin of victory [12], the scoring first [13], and the differences in playing styles across hemispheres [14,15,16], these aspects should be considered in a data mining investigation.

Data mining is a process of extracting and discovering patterns in datasets. Sports data mining assists coaches and managers in result prediction, player performance assessment, player injury prediction, sports talent identification, and game strategy evaluation [17]. In particular, decision tree (DT) is a machine learning technique that applies an approach of dividing data into smaller clusters to identify patterns that can be used for prediction. The logical structure consists in a hierarchical combination of decisions from the root to the terminal (i.e., leaf) nodes, and these provide knowledge based on the classification. Exhaustive CHAID (i.e., Chi-squared Automatic Interaction Detector) method is used based on the chi-square test of association. An Exhaustive CHAID tree is a decision tree constructed by repeatedly splitting subsets of the space into two or more child nodes, beginning with the entire data set, until only two super categories are left. Exhaustive CHAID can find the best split for each predictor variable by minimize the variation within nodes in order to construct homogenous subgroups in the decision tree diagram [18]. Decision trees are usually assessed for accuracy by means of cross-validation techniques [19]. In particular, dataset is randomly subsampled and is entirely used both for training and validation, maximizing the dataset volume.

In the last decade HA was extensively studied in several sports [1] and classification decision trees were used to assess the effect of performance indicators on game outcome in rugby league [20,21] as well as in rugby union [22,23]. However, to the best of our knowledge, this is the first study to apply a flexible and nonlinear statistical model to investigate HA in professional rugby union. In particular, the aim of the present study was to analyze the HA of professional high-level rugby club competition from a complex dynamical system perspective according to the tournament, the margin of victory, the scoring first, and the crowd support.

## 2. Materials and Methods

### 2.1. Design

This study comprised 7934 performances (3967 games) played by professional teams from elite national (i.e., English Premiership = 1824 (23%), French Top14 = 2520 (32%), Currie Cup = 566 (7%), Mitre 10 Cup = 1188 (15%)) and international (i.e., Pro14 = 1836 (23%)) competitions during the last 6 competitive seasons before (2013/14 to 2018/19) and after (2020/21) the COVID-19 pandemic period. The 2019/20 season was excluded from the analysis because of the irregular and intermittent game schedule. Archival data were collected by a researcher from the Ultimate Rugby web domain (https://www.ultimaterugby.com/# (accessed on 16 July 2021)). Data reported in this Web domain were collected by a researcher and stored into a .csv file. The local institutional review board approved this study.

### 2.2. Methodology

Win and lose but not drawn performances were considered in this study. For each of the 7938 performances fixture (home vs. away), outcome (win vs. lose), margin of victory [√(points scored-points conceded)^2^], season (Pre-COVID vs. COVID 20–21), tournament (Pro14 vs. Top14 vs. Premiership vs. Currie Cup vs. Mitre 10 Cup), and the first event of the game (scoring, missed scoring, yellow or red card, and substitution) were considered. In particular, scoring included scored try with and without conversion, penalty try, and kick at goal, while missed scoring included missed penalty (i.e., kick at goal failed). Noting the relation of the margin of victory (i.e., final score difference) [12,24] and of the first scoring [20] with the relative success of the game plan adopted by the winning teams, the same were included in this study. The margin of victory was clustered within the decision tree to define 3 clusters (closed, balanced, and unbalanced games).

### 2.3. Data Analysis

An exhaustive chi-square automatic interaction detection (CHAID) decision tree was grown using win/lose as the binary response variable in IBM SPSS Statistics package (version 27, IBM Corp., New York, NY, USA) using a ten-fold cross validation. Outcome was set as dependent variable while season, fixture, margin of victory, tournament, and first event were set as independent variables. In order to manage both accuracy and complexity of the model (i.e., the maximum tree depth, which contains the highest value of accuracy, is five), grow limits was set to 5 levels and minimum number of cases for parent and child node was set at 100 and 50, respectively. Level of significance for splitting nodes was set at *p* ≤ 0.05 and within multiple comparisons, significance values for merging and splitting criteria were adjusted using the Bonferroni method. The intervals for the continuous variable (i.e., margin of victory) was set at 3, corresponding to the closed, balanced, and unbalanced games clusters.

## 3. Results

**Figure 1:**
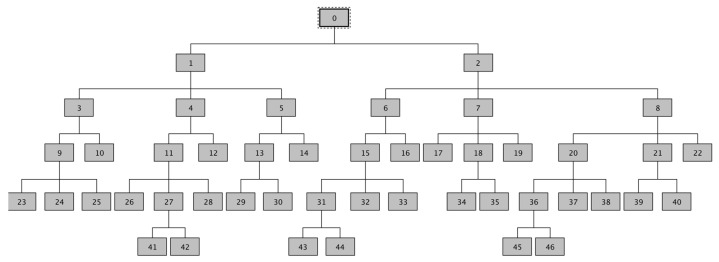
Decision Tree (DT) Diagram.

**Figure 2:**
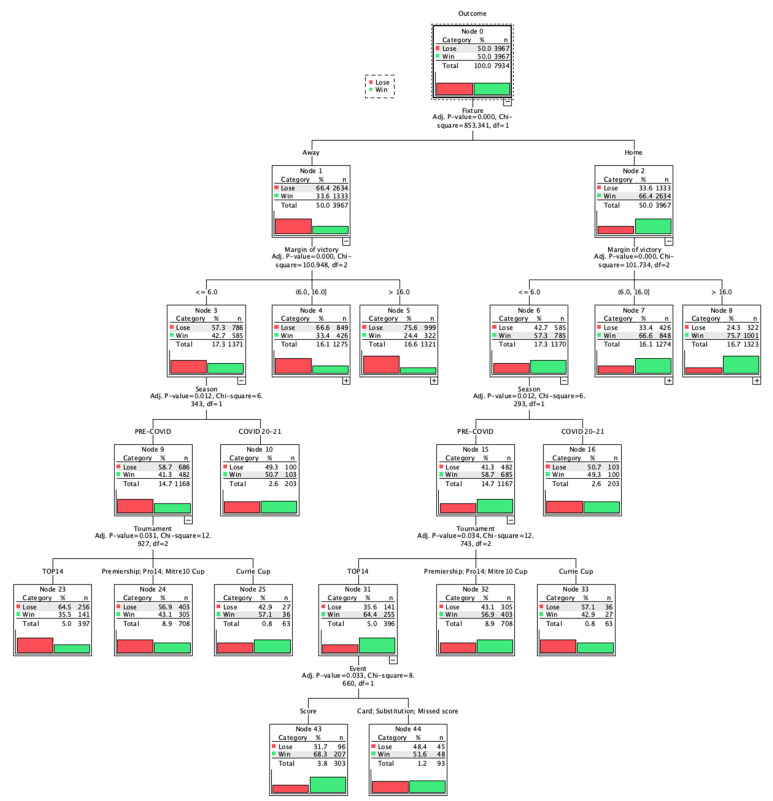
Closed Games Diagram from the Away and Home Fixture Perspective.

Out of the 7934 performances, the model successfully classified 2658 (67.0%) of the 3967 loses and 2663 (67.1%) of the 3967 wins. The model has grown 47 nodes within all the 5 levels and 28 leaves (i.e., terminal nodes). The diagram and the detailed table of the entire model are presented in Figure 1 and in Table S1, respectively. Figure 2 resumes closed games both from the away and home fixture perspective.

From the fixture perspective (node 1–2) teams playing at home were 66.4% likely to win the game. At the second level of depth (nodes from 3 to 8), the values of the margin of victory were divided into 3 clusters, below 6 points for closed, from 6 to 16 for balanced, above 16 for unbalanced games, respectively.

From closed games in home fixture perspective (node 6) the crowd absence affected HA by 9.4% (49.3% vs. 58.7%). At a deeper level, during the Pre-COVID period, the HA was significantly higher for Top14 championship (node 31) compared to other championships (64.4% vs. 56.9% vs. 42.9%). In Top14, HA was higher when scoring was the first event of the game. From balanced games in home fixture perspective (node 7), the highest HA was in Top14 championship (node 17) compared to the others (74.1% vs. 65.9% vs. 53.6%). In Premiership, Pro14, and Mitre 10 Cup (node 18) crowd absence affected HA by 16.2% (68.2% vs. 52%). From unbalanced games in home fixture perspective (node 8), HA was higher (79.8% vs. 45.6% vs. 69.8%) when the first event of the game was scoring (node 20) compared to card or substitution (node 21) and to missed score (node 22), respectively. When scoring first, HA was highest in Top14 (node 36) compared to the others (89.2% vs. 77.8% vs. 70.6%). In Top14 (node 36), crowd absence affected HA by 10.8% (90.2% vs. 80%). When the first event was card or substitution in Currie Cup or Premiership or Mitre 10 Cup (node 40), HA was lower than the others (31.1% vs. 59.4%). Figure 2, Figures S1 and S2 present the decision tree for home fixture according to closed, balanced and unbalanced games, respectively.

In closed games in away fixture perspective (node 3), results are specular to those in closed games in home fixture perspective (node 6). From balanced games in away fixture perspective (node 4), HA was higher (37.5% vs. 23%) when the first event of the game was scoring (node 11) compared to card or substitution or missed score (node 12), respectively. At a deeper layer, the highest HA (49.6%) was reported in Mitre 10 Cup (node 28) compared to Premiership or Pro14 or Currie Cup (node 27) and Top14 (node 26) when scoring first. Scoring first in Premiership or Pro14 or Currie Cup without crowd support increases HA by 17.1% (54.5% vs. 37.4%). From unbalanced games in away fixture perspective (node 5), HA was higher (30% vs. 12.2%) when the first event of the game was scored (node 13) compared to card or substitution or missed score (node 14), respectively. At a deeper layer, the highest HA (36.4%) was reported in Mitre 10 Cup, Premiership, Pro14, or Currie Cup (node 30) compared to Top14 when scoring first occurred (node 29). Figure 2, Figures S3 and S4, present the decision tree for away fixture according to closed, balanced and unbalanced games, respectively.

## 4. Discussion

Since HA was reported to be influenced by the crowd support, the aim of the present study was to analyze it considering the crowd absence during the unique COVID-19 pandemic situation. Thus, HA was investigated within professional rugby club competitions according to the tournament, the margin of victory, and the scoring first. The main findings of this study were that the HA disappeared when competing without the supporters, especially in closed games, where differences in the final score were less than a converted try (7 points). Although differences between tournaments emerged, the crowd absence was associated with a detrimental effect on HA in both northern and southern hemisphere competitions.

From a complex system perspective, non-linear approaches for clustering and interpreting high-dimensional datasets, as Self Organizing Maps and Decision trees, were used in rugby union performance analysis [22,25,26] in order to better understand the determinants of success. To date, linear statistical models (e.g., analysis of variance, linear regression, chi-square) are the most common statistical tools used in analyzing HA in rugby union [1,4,7] although the use of non-linear statistics and machine learning techniques were shown to be a powerful and robust tool in detecting the most influent independent variables within large samples [11,27]. In fact, from a multivariate perspective, supervised non-linear statistical modeling technique like Exhaustive CHAID decision tree (i.e., Chi-squared Automatic Interaction Detector) can handle both nominal and numeric input variables, it is capable of handling datasets that may have errors, outliers, and missing values, and is considered to be a nonparametric method [28,29]. Moreover, its representation is easy to follow and it can be comprehensible by non-professional users [28,29]. On the other hand, it can be subject to overfitting and underfitting, particularly when using a small data set and this effect could limit the robustness of the model. Finally, strong correlation between different potential independent variables may improve the model statistics even if they are not causally related to the dependent variable. Therefore, projecting and interpreting DT models should consider these pros and cons [28,29], taking into account that adding multiple contextual variables in a non-linear perspective could enhance insight in performance analysis in rugby [30] and help coaches and coaching staff to better identify opportunities and threats. In this study a rather complex DT was grown, but it can be made simpler by following each variable of interest at a time.

With respect to the variables within the DT, the margin of victory had the highest impact on the classification (i.e., 2nd level of depth) and hence three clusters were built (i.e., ≤6, 6–16, >16). Compared to previous studies [12,24] in rugby union, the cutoff values in this study were lower. In fact, closed games were considered when differences in the final score were less than a converted try (i.e., <7 points), compared to 9 [12] and to 15 or 11 [24]. In this situation the HA was significantly lower than in balanced and unbalanced games, stressing the higher outcome uncertainty that was previously reported in rugby union [12,24].

When differences between teams are minimal and the outcome uncertainty is high, like in closed games, alterations in contextual variables can be substantial. In fact, contextual differences caused by COVID-19 pandemic had a significant influence on the game outcome exclusively in closed games. In particular, the crowd absence negatively influenced the HA, reducing it from 58% to less than 50% (i.e., 49.3%). Although the crowd absence had an effect even in balanced and unbalanced games, it resulted less important to the differences between tournaments and to the first event of the game. The scenario induced by the COVID-19 pandemic was detrimental for the HA in any case, even when it was secondary to other variables (i.e., nodes 34, 35, 41, 42, 45, 46), and it altered HA progressively less in closed, balanced and unbalanced games, respectively (i.e., nodes 6, 7, 8).

Differences between tournaments emerged when describing HA in rugby union. In fact, HA was always higher in Top14 compared to other tournaments, providing higher chances of winning without distinction for closed, balanced, and unbalanced games. Moreover, for teams playing away in Top14 in balanced and unbalanced games, scoring first does not represent a significant advantage compared to other tournaments (nodes 26, 29), like in Mitre 10 Cup where scoring first in balanced games nullifies HA (node 28). On the contrary, teams playing home in unbalanced games in Top14, as well as in Pro14, maintained HA even when they received a penalty card or they made a substitution at the very start of the game (i.e., 59.4%), unlike it happened for other tournaments (i.e., 31% in Premiership, Mitre 10 Cup, and Currie Cup). In the Pre-COVIDperiod, HA in Top14 was less affected by negative contextual variables (i.e., penalty cards or early substitutions), especially in unbalanced games (i.e., node 39). Based on several key performance indicators, it was suggested that playing style in Top14 is characterized by very few opportunities to spread the ball wide and to play a fast-paced game [31]. In addition, French Top14 is one of the oldest and more successful championship in the northern hemisphere in terms of attendance [32], as well as one of the highest paid rugby domestic league [33] attracting many elite foreign players. These characteristics could have made Top14 more resilient in terms of HA, less sensitive to negative contextual variables and more sensitive to positive ones (i.e., scoring first).

In general, HA was also modulated by the first event of the game. In rugby league scoring first was reported to increase chances of success [13] and this phenomenon would be in line also with rugby union. Although it was not investigated in rugby union before, in this study scoring first enhanced HA especially in balanced and unbalanced games, for both teams playing away and at home (i.e., nodes 11, 13, 20). Conversely, negative events like receiving a penalty card or making an early substitution reversed the HA for teams playing home (i.e., node 21) and penalized even more teams playing away (i.e., nodes 12, 14). Even if success in rugby union is multifactorial phenomenon depending on technical and tactical and time-motion events [12,34], the first event of the game should be take into account for estimating the outcome of the game.

## 5. Conclusions

COVID-19 pandemic situation stressed the importance of the crowd support in rugby union elite competitions. HA was influenced by the absence of the crowd support, although it should be considered as a multifactorial phenomenon depending on several variables. Considering the margin of victory, closed games are more sensitive to contextual variables in altering HA with respect to balanced and unbalanced games. Differences in HA depend on the tournament also, notably in the Top14 where playing home adds significant advantage to winning games compared with all the other tournaments. In their turn, all the above-mentioned changes in HA are sensitive to the first technical and tactical event of the game. Similar to rugby league, scoring first increases HA while receiving a penalty card decreases it in rugby union also, especially in balanced and unbalanced games.

This study is in line with others that investigated performance analysis by means of the decision tree classification method, albeit no cut-off is set for the classification validity [21,35]. Moreover, because of the advantages of the non-linear statistics (i.e., such as decision trees) in terms of ability to cope with errors, outliers, and missing values within databases, and ease of understanding by non-professional users, they should be preferred when describing multidimensional complex scenarios in performance analysis. Finally, further investigation on the characteristics of the tournaments (e.g., physical status, relative age effect, presence of top players) should be undertaken to better explain HA.

## Data Availability

Not applicable.

## Supplementary Materials

The following are available online at https://www.mdpi.com/article/10.3390/ijerph182312711/s1, Figure S1: Home balanced games, Figure S2: Home unbalanced games, Figure S3: Away balanced games, Figure S4: Away unbalanced games, Table S1. EXHAUSTIVE CHAID decision tree table.
