# Supplementary material for: A Machine Learning Approach to Analyze Home Advantage during COVID-19 Pandemic Period with Regards to Margin of Victory and to Different Tournaments in Professional Rugby Union Competitions
Source: Int J Environ Res Public Health. 2021 Dec 2;18(23):12711. doi: 10.3390/ijerph182312711 (PMC8656876; doi:10.3390/ijerph182312711)
Supplement: Supplementary file 1 [file ijerph-18-12711-s001.zip › ijerph-1451485-supplementary.pdf]

International Journal of
Environmental Research and Public Health

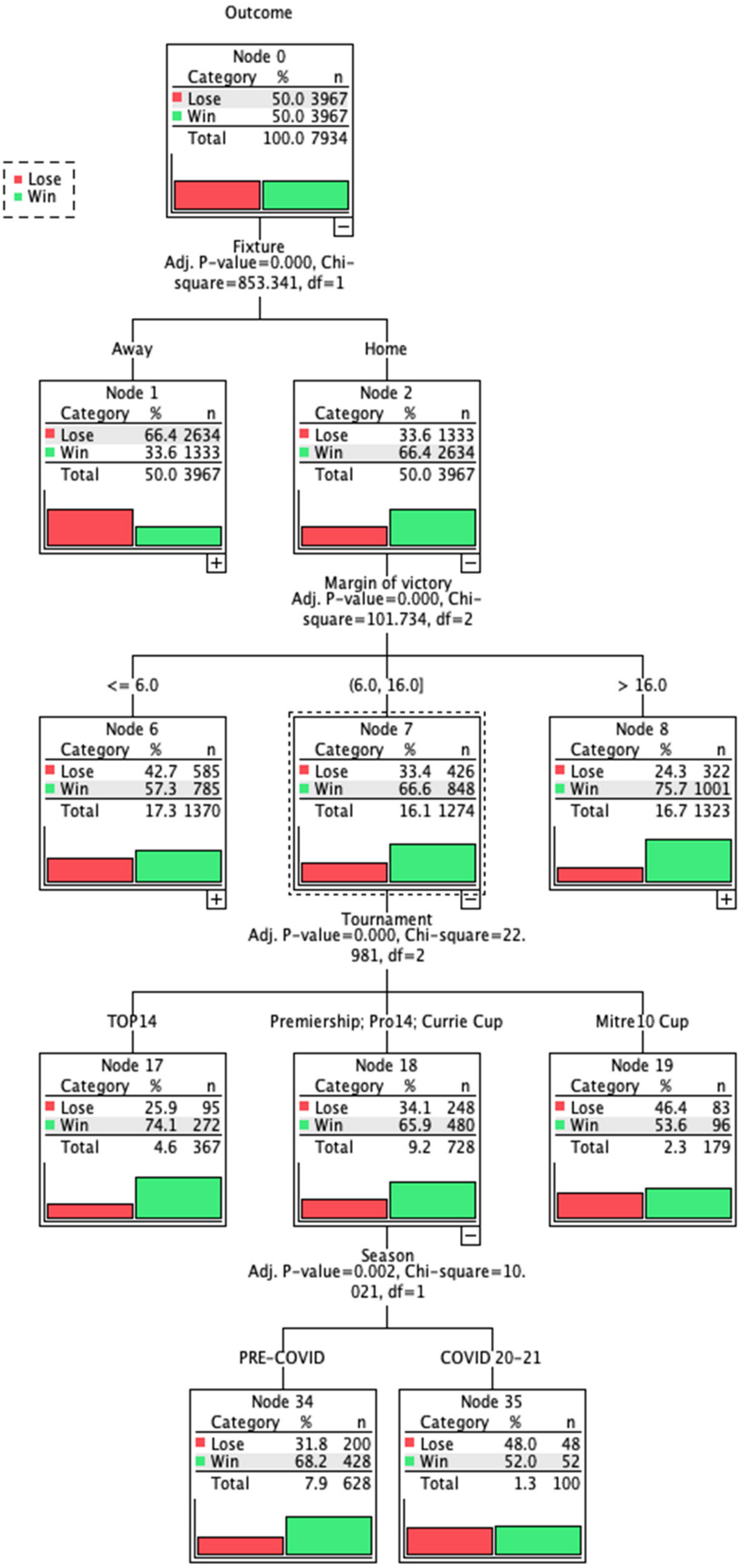

Figure S1. Home Balanced Games.

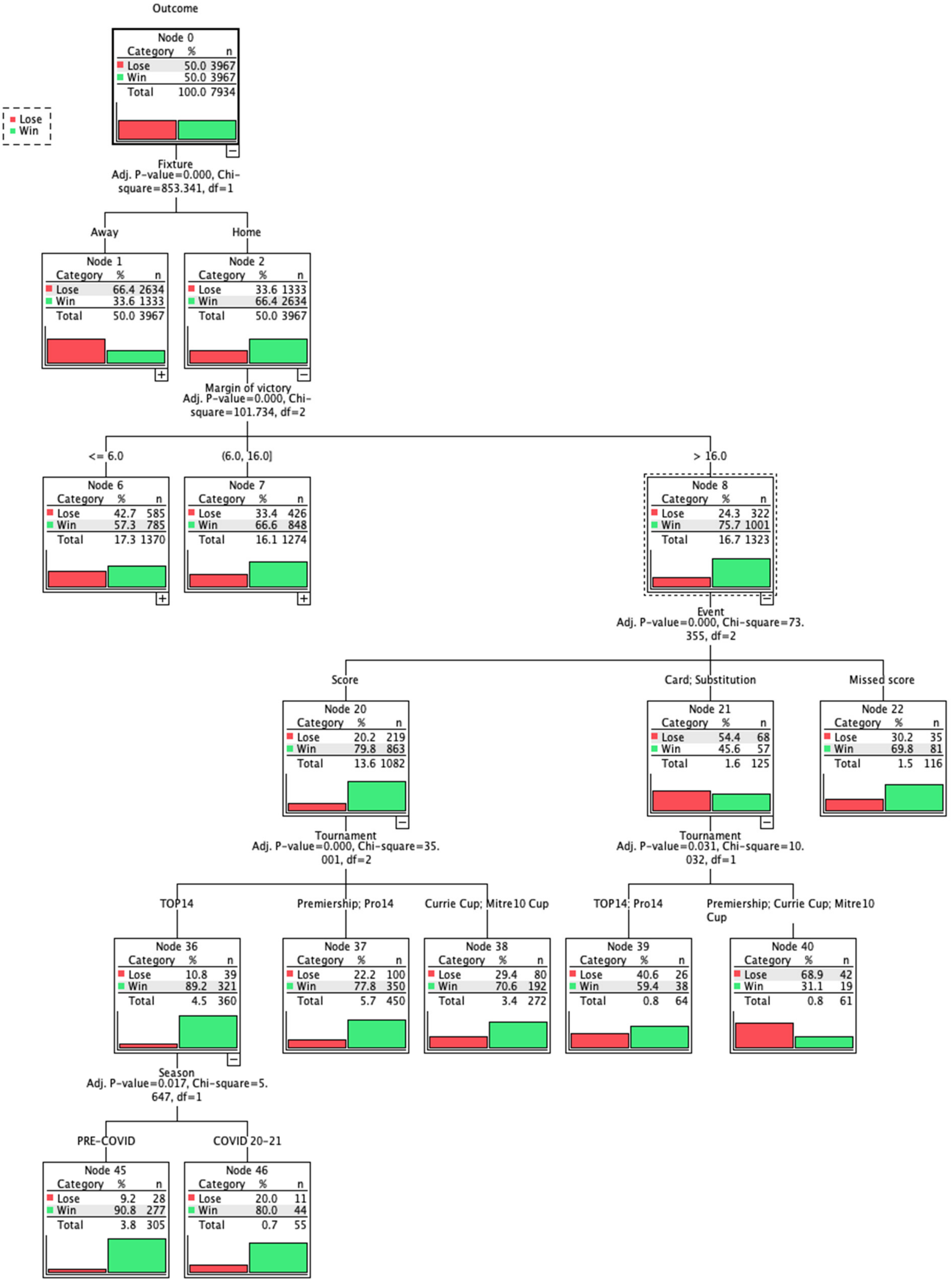

Figure S2. Home Unbalanced Games.

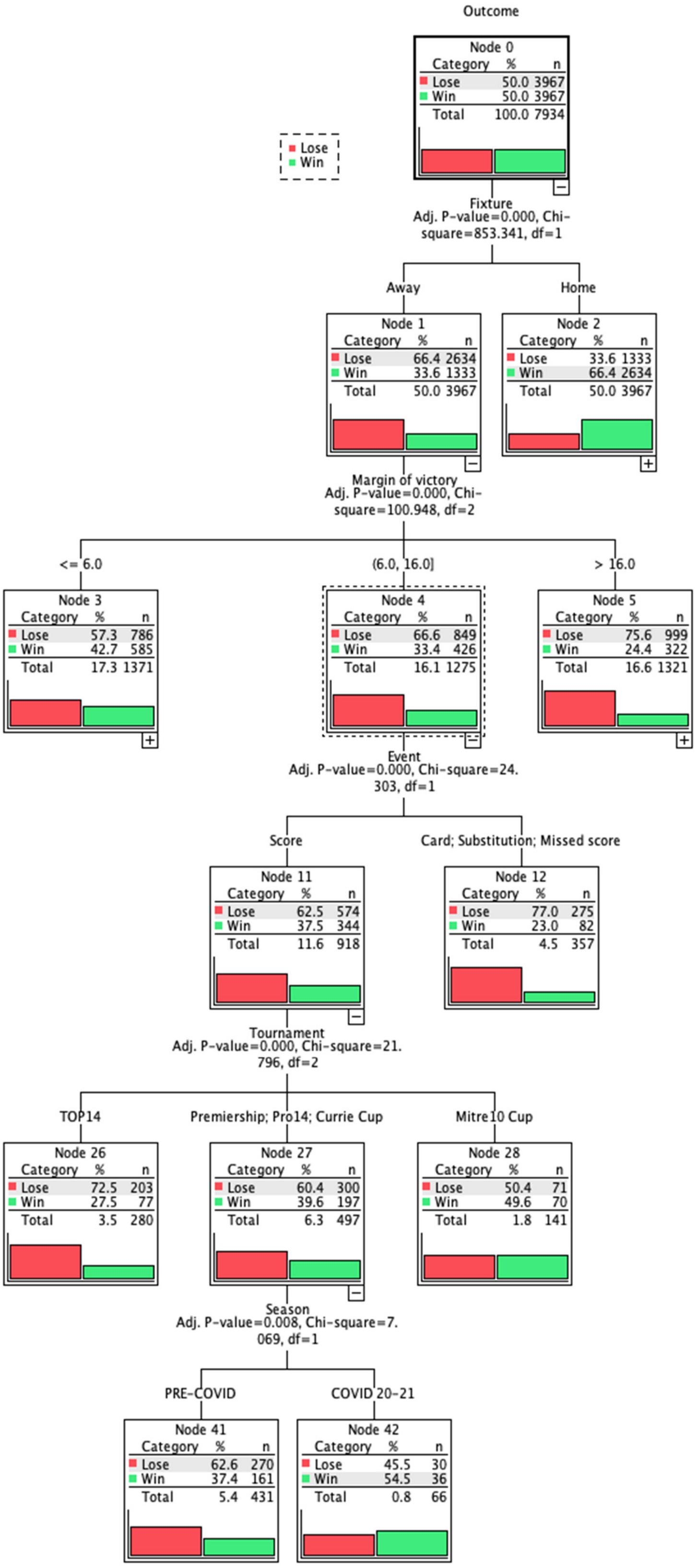

Figure S3. Away Balanced Games.

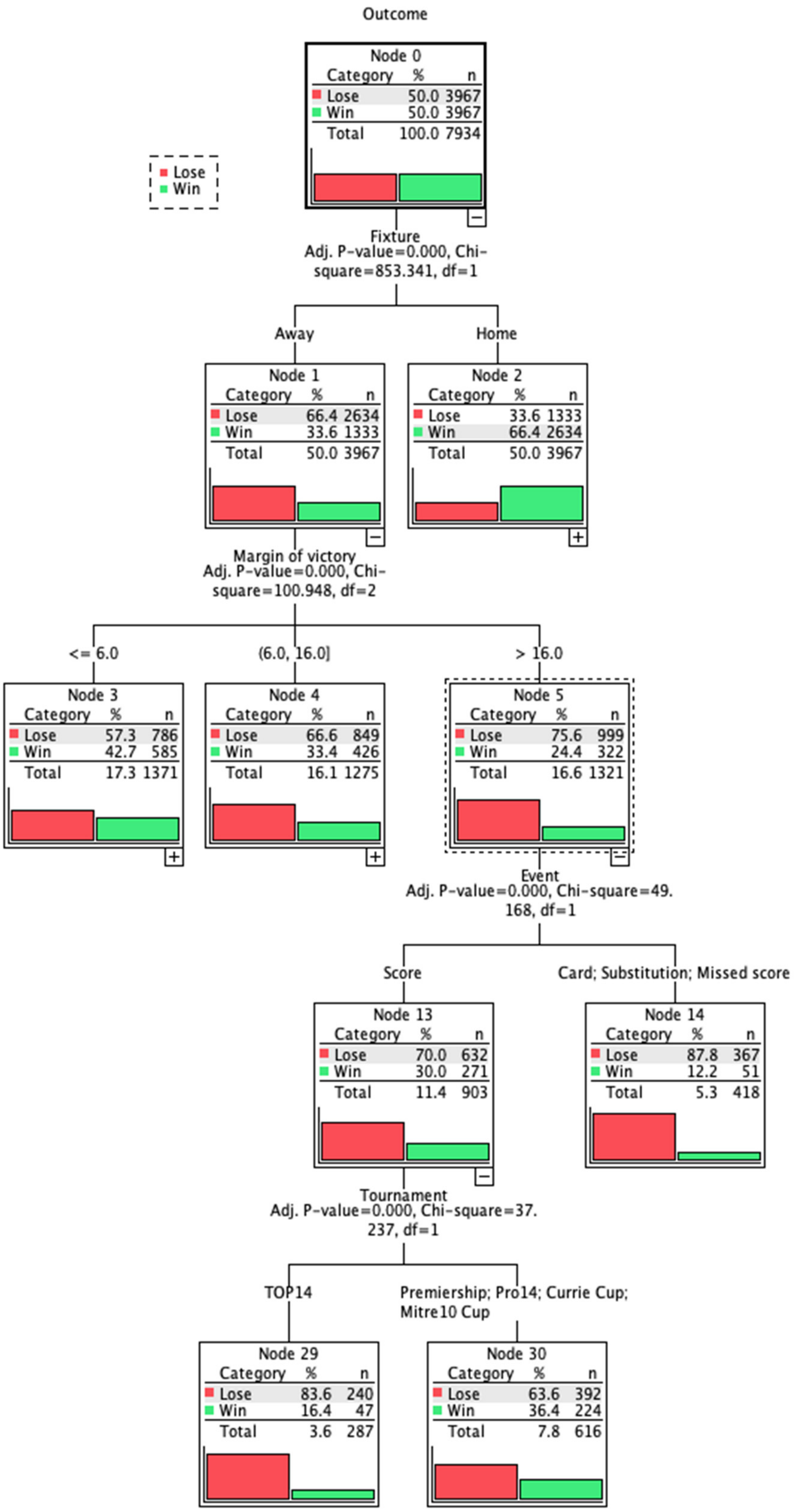

Figure S4. Away Unbalanced Games.

Table S1. EXHAUSTIVE CHAID Decision Tree Table.

Node	Lose		Win		Total		Predicted Category	Parent Node	Primary Independent Variable				
	N	Percent	N	Percent	N	Percent			Variable	Sig.[a]	Chi-Square	df	Split Values
0	3967	50.0%	3967	50.0%	7934	100.0%	Lose						
1	2634	66.4%	1333	33.6%	3967	50.0%	Lose	0	Fixture	0.000	853.341	1	Away
2	1333	33.6%	2634	66.4%	3967	50.0%	Win	0	Fixture	0.000	853.341	1	Home
3	786	57.3%	585	42.7%	1371	17.3%	Lose	1	Margin of victory	0.000	100.948	2	≤ 6.0
4	849	66.6%	426	33.4%	1275	16.1%	Lose	1	Margin of victory	0.000	100.948	2	(6.0, 16.0]
5	999	75.6%	322	24.4%	1321	16.6%	Lose	1	Margin of victory	0.000	100.948	2	> 16.0
6	585	42.7%	785	57.3%	1370	17.3%	Win	2	Margin of victory	0.000	101.734	2	≤ 6.0
7	426	33.4%	848	66.6%	1274	16.1%	Win	2	Margin of victory	0.000	101.734	2	(6.0, 16.0]
8	322	24.3%	1001	75.7%	1323	16.7%	Win	2	Margin of victory	0.000	101.734	2	> 16.0
9	686	58.7%	482	41.3%	1168	14.7%	Lose	3	Season	0.012	6.343	1	PRE-COVID
10	100	49.3%	103	50.7%	203	2.6%	Win	3	Season	0.012	6.343	1	COVID 20-21
11	574	62.5%	344	37.5%	918	11.6%	Lose	4	Event	0.000	24.303	1	Score
12	275	77.0%	82	23.0%	357	4.5%	Lose	4	Event	0.000	24.303	1	Card; Substitution; Missed score
13	632	70.0%	271	30.0%	903	11.4%	Lose	5	Event	0.000	49.168	1	Score
14	367	87.8%	51	12.2%	418	5.3%	Lose	5	Event	0.000	49.168	1	Card; Substitution; Missed score
15	482	41.3%	685	58.7%	1167	14.7%	Win	6	Season	0.012	6.293	1	PRE-COVID
16	103	50.7%	100	49.3%	203	2.6%	Lose	6	Season	0.012	6.293	1	COVID 20-21
17	95	25.9%	272	74.1%	367	4.6%	Win	7	Tournament	0.000	22.981	2	TOP14
18	248	34.1%	480	65.9%	728	9.2%	Win	7	Tournament	0.000	22.981	2	Premiership; Pro14; Currie Cup
19	83	46.4%	96	53.6%	179	2.3%	Win	7	Tournament	0.000	22.981	2	Mitre10 Cup
20	219	20.2%	863	79.8%	1082	13.6%	Win	8	Event	0.000	73.355	2	Score
21	68	54.4%	57	45.6%	125	1.6%	Lose	8	Event	0.000	73.355	2	Card; Substitution
22	35	30.2%	81	69.8%	116	1.5%	Win	8	Event	0.000	73.355	2	Missed score
23	256	64.5%	141	35.5%	397	5.0%	Lose	9	Tournament	0.031	12.927	2	TOP14
24	403	56.9%	305	43.1%	708	8.9%	Lose	9	Tournament	0.031	12.927	2	Premiership; Pro14; Mitre10 Cup
25	27	42.9%	36	57.1%	63	0.8%	Win	9	Tournament	0.031	12.927	2	Currie Cup
26	203	72.5%	77	27.5%	280	3.5%	Lose	11	Tournament	0.000	21.796	2	TOP14
27	300	60.4%	197	39.6%	497	6.3%	Lose	11	Tournament	0.000	21.796	2	Premiership; Pro14; Currie Cup
28	71	50.4%	70	49.6%	141	1.8%	Lose	11	Tournament	0.000	21.796	2	Mitre10 Cup
29	240	83.6%	47	16.4%	287	3.6%	Lose	13	Tournament	0.000	37.237	1	TOP14
30	392	63.6%	224	36.4%	616	7.8%	Lose	13	Tournament	0.000	37.237	1	Premiership; Pro14; Currie Cup; Mitre10 Cup
31	141	35.6%	255	64.4%	396	5.0%	Win	15	Tournament	0.034	12.743	2	TOP14
32	305	43.1%	403	56.9%	708	8.9%	Win	15	Tournament	0.034	12.743	2	Premiership; Pro14; Mitre10 Cup
33	36	57.1%	27	42.9%	63	0.8%	Lose	15	Tournament	0.034	12.743	2	Currie Cup
34	200	31.8%	428	68.2%	628	7.9%	Win	18	Season	0.002	10.021	1	PRE-COVID
35	48	48.0%	52	52.0%	100	1.3%	Win	18	Season	0.002	10.021	1	COVID 20-21
36	39	10.8%	321	89.2%	360	4.5%	Win	20	Tournament	0.000	35.001	2	TOP14
37	100	22.2%	350	77.8%	450	5.7%	Win	20	Tournament	0.000	35.001	2	Premiership; Pro14
38	80	29.4%	192	70.6%	272	3.4%	Win	20	Tournament	0.000	35.001	2	Currie Cup; Mitre10 Cup

39	26	40.6%	38	59.4%	64	0.8%	Win	21	Tournament	0.031	10.032	1	TOP14; Pro14
40	42	68.9%	19	31.1%	61	0.8%	Lose	21	Tournament	0.031	10.032	1	Premiership; Currie Cup; Mitre10 Cup
41	270	62.6%	161	37.4%	431	5.4%	Lose	27	Season	0.008	7.069	1	PRE-COVID
42	30	45.5%	36	54.5%	66	0.8%	Win	27	Season	0.008	7.069	1	COVID 20-21
43	96	31.7%	207	68.3%	303	3.8%	Win	31	Event	0.033	8.660	1	Score
44	45	48.4%	48	51.6%	93	1.2%	Win	31	Event	0.033	8.660	1	Card; Substitution; Missed score
45	28	9.2%	277	90.8%	305	3.8%	Win	36	Season	0.017	5.647	1	PRE-COVID
46	11	20.0%	44	80.0%	55	0.7%	Win	36	Season	0.017	5.647	1	COVID 20-21

Growing Method: EXHAUSTIVE CHAID

Dependent Variable: Outcome

a. Bonferroni adjusted
